# A simple method for primary screening of antibacterial peptides in plant seeds

**Published:** 2011-06

**Authors:** A Aliahmadi, R Roghanian, G Emtiazi, A Ghassempour

**Affiliations:** 1 Department of Biology, Faculty of Science, University of Isfahan. Iran; 2 Department of Biology, Medicinal Plants and drug Research Institute, Shahid Beheshti University, Tehran, Iran; 3 Department of Phytochemistry, Medicinal Plants and drug Research Institute, Shahid Beheshti University. Tehran. Iran

**Keywords:** Plant antibacterial peptides, *Staphylococcus aureus*, *Enterococcus faecium*, screening method

## Abstract

**Background and Objectives:**

Regarding the importance of finding new antibacterial drugs, screening of plants as a promising resource are now conducted worldwide. In this study, we report the application of a simple previously described method for screening of different plant seeds in order to find the best resources of plant antimicrobial peptides.

**Materials and Methods:**

Total water soluble protein of 10 different plant seeds were extracted and subjected to SDS-PAGE and subsequent agar-overlay bioassays. Standard strains of *Staphylococcus aureus*, *Enterococcus faecium* and *Escherichia coli* were included in the bioassays. This method also was used for total proteins precipitated by Ammonium sulphate which ensure the protein nature of the test substances. Molecular size and the amounts of effective peptides were estimated using Tricin-SDS-PAGE and densitometry.

**Results:**

Two different plant seeds showed noticeable antibacterial activities against tested Gram positive bacteria and a moderate inhibitory effect on Gram negative ones. Based on the results of Tricin-SDS-PAGE analysis which were carried out in parallel to bioassays, it was concluded that effective antibacterial substances are peptides with molecular weight of slightly larger than 5 kDa.

**Conclusion:**

On the basis of results of agar-overlay experiments and by screening of 10 different herbal seeds, we could introduce seeds of *M. sativa* L. and *Onobrychis sativa* Lam., as great sources of putative plant antibacterial peptides. The proposed screening method can be used for screening of large number of different plant seeds and even other parts of the plant body, regarding some necessary modification in total water soluble protein extraction steps.

## INTRODUCTION

There is a progressively increasing global attention being paid to defeat multi-drug resistant bacterial infections (1) and plants are amongst promising resources for finding of new antibacterial agents (2). Besides phenols, alkaloids and some plant secondary metabolites with known antimicrobial properties, and plant antimicrobial peptides could also be beneficial for this purpose (3, 4). These antimicrobial agents are very similar to those of human antimicrobial peptides in structure and function and are produced as a part of their defence systems (4). Most of these are small Cystein-rich basic peptides which have been also classified as Cationic Antimicrobial Peptides (CAMPs) in all living organisms (5). As reported elsewhere, these antimicrobial peptides are now known as good antifungal substances and occasionally have antibacterial activities *in vitro* (3, 4). Purification and characterization of these peptides is now conducted worldwide and different parts of plants have been subjected to such investigations (6). According to reported studies, purification of antimicrobial peptides is a time consuming and relatively expensive process carried out generally with several chromatography steps and parallel antimicrobial assays (7, 8). However, application of an easy and cost benefit screening method which could help to analyse a large number of plant samples regarding their antimicrobial peptides, would be helpful for finding of better plant resources for new therapeutic agent candidates. Here we report the application of a previously introduced method for bacteriocin production assay (9) as simple and efficient way for screening and primary identification of antimicrobial peptides in plant seeds.

## MATERIALS AND METHODS

**Bacterial strains.** *Staphylococcus aureus* ATCC 25923, *Enterococcus faecium* TX100, *Escherichia coli* ATCC 25922.

**Plant material.** The plant seeds were purchased from Pakan Bazr (Isfahan, Iran) and these included *Medicago sativa* L., *Onobrychis sativa* Lam., *Trifolium repens* L.*, Vicia vilosa* Roth.*, Artemisia sieberi* Besser.*, Secale cereale* L.*, Astragalus* L.*, Avena sativa* L.*, Stipa barbata* Desf. and *Hordeum bolbosum* L.

**Water soluble protein extraction from seeds.** Uniform seeds of each plant were selected, rinsed with water and Distilled Water (DW) and dried under a chemical hood. Milled seeds were subjected to total protein extraction using extraction buffer containing 50 mM phosphate buffer (pH 7), 2 mM EDTA, 5% glycerol and 50 mM NaCl. Cold extraction buffer was added to the milled seeds (10:1, V/W) and the mixture was put on a shaker for up to 2h at 4°C. Centrifugation was done at 12,000 rpm for 20 min at 4°C and the clarified protein solutions were passed through sterile gauze and then stored at −20°C.

**SDS**-**PAGE and Tricine-SDS**-**PAGE analysis.** Concentration of clarified protein samples were assessed by standard protocol (10). From every seed, different amounts of total water soluble proteins were mixed with sample buffer containing 2 Mercaptoethanol (2ME) as reducing agent and heated for 5 minutes at 100°C and then run onto 15% SDS-PAGE with constant voltage of 100V. Each electrophoresis experiment was terminated when the tracking dye reached 1 cm above the sealing part of the gel cast.

Tricine-SDS gels were also used for higher resolution of low molecular weight protein and peptides. This was necessary for detection of the relative molecular mass of the effective substance and for estimation of the amount of effective peptides by the densitometer (11). 50 µg of samples were used. Coomassie brilliant blue staining method was used for visualisation of protein bands, and Insulin (molecular weight of about 6 kDa) was applied as low molecular weight protein marker.

**Agar**-**overlay assay. **SDS containing gels were subjected to agar overlay as described previously (9). Briefly, each gel slice was fixed for 2 hours by using a fixation solution containing 20% isopropanol, 10% acetic acid in sterile DW and then was washed by about 300 ml of sterile DW for additional 3h at room temperature (6 times, 50 ml each). Bacterial strains were grown overnight on Nutrient agar plates at 35°C. Each washed gel was placed on a sterile Petri dish and 7 ml of 50°C Mueller-Hinton agar medium containing 0.75% agar and 5×10^7^ CFU of the test microorganism was poured on the gel. Incubation was done at 35°C for 20 h.

A SDS gel was also run just with the sample buffer and DW as negative control for investigation of the probable effects of sample buffer components or trace amounts of remaining SDS in antimicrobial assays for each tested microorganism. This gel also was fixed and washed according to the above described protocol.

**Agar overlay bioassay using Ammonium sulphate precipitated proteins and peptides**. In the case of *M. sativa* L. and *Onobrychis sativa* Lam. which exhibited the best results in initial agar overlay assay, total water soluble protein and peptides of seeds were precipitated using Ammonium sulphate (up to 80% saturation) and then the precipitated materi-als were subjected to agar overlay assay as described above. This step was done to ensure preservation of the protein nature of active antibacterial substances.

## RESULTS

**Fig. 1:**
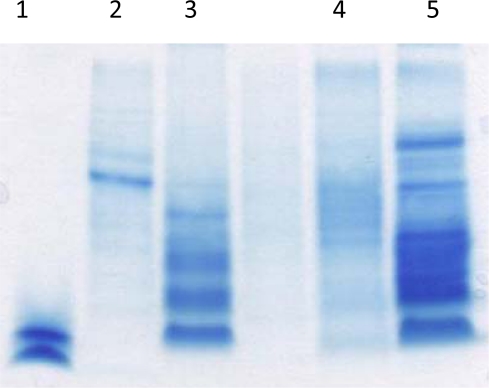
Tricine-SDS PAGE analysis of some plant seed total water soluble proteins (about 40-50µg of each sample according to Bradford assay). 1. Insulin as LMW marker oF 40-50µg of each sample according to Bradford assay). 1. Insulin as LMW marker

**Fig. 2:**
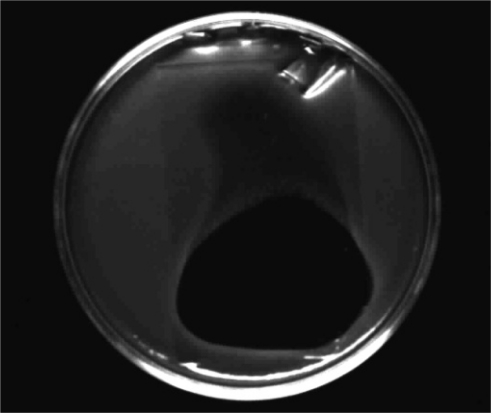
Activity of *M. sativa* total water soluble proteins against *S. aureus* strain in agar overlay assay (about 20 µg of effective peptides according to data of densitometry).

**Fig. 3:**
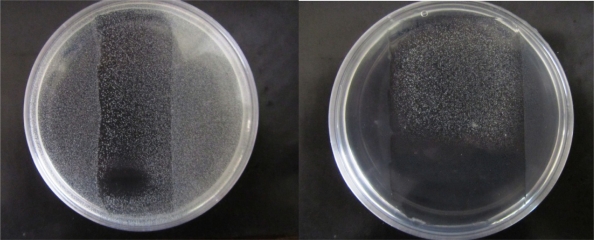
Activities of *M. sativa* total water soluble proteins against *E. faecium* strain in agar overlay assays. Left plate shown the effect of about 5 µg of putative peptides and right one is the effect of about 20 µg of them.

As shown in Fig. 1, some tested seeds gave dense regions of low molecular weight proteins and peptides in Tricine-SDS gels. In agar-overlay assays, there were clear and noticeable zones of inhibition in the case of tested gram positive bacteria (Fig. 2 & 3), but a smaller zone with several colonies against *E. coli* strain for two screened plant seeds. According to the location of the inhibitory zone in the agar overlay assay, we concluded that there are some antibacterial peptides in the two mentioned plant seeds.

When 50 µg samples of total water soluble proteins and peptides were applied onto SDS containing gels, *Medicago sativa* L. and *Onobrychis sativa* Lam. seeds showed the most powerful antibacterial activities against both *E. faecium* and *S. aureus* strains respectively. As indicated in Fig. 3, in the case of *M. sativa* and *E. faecium*, there was a noticeable increase in zone of inhibition when the amount of effective peptides was increased by four times. The relative size of inhibition zones were different when two gram positive bacteria were assessed against the two mentioned plant seeds proteins and overall inhibitory effects of both samples were higher against *E. faecium* than *S. aureus* strain according to data from three fully separated experiments (total water soluble protein preparation, SDS-PAGE and subsequent agar overlay assays). When ammonium sulphate precipitated materials were used as test sample in agar overlay bioassay, inhibition zones were decreased slightly which may have been caused by errors in determination of protein concentration.

There was not any zone of inhibition when the agar overlay assays performed. So intensive washing by mentioned fixation solution and sterile D.W. could remove any interfering SDS as well.

## DISCUSSION

Production of antimicrobial peptides; also known as defensins in some literatures, is a common feature of all living organisms from bacteria to humans (4, 5, 9). There are many reports on identification, characterization and even biotechnological application of plant defensins especially as an approach against plant pathogens (12, 13). Plant defensins not only have been introduced as anti-fungal candidates, but also are being studied recently for their antibacterial properties (12) and in the case of human pathogens, they could be promising candidates especially against gram positive bacteria on the basis of known biological activity of other similar antimicrobial peptides isolated from human or bacteria (5, 9).

Here, we introduce a simple screening method for identification of plant seeds having antibacterial peptides. Most of reported studies on plant defensins include time-consuming procedure of purification and antimicrobial activity evaluation of purified materials (13, 14). However any precise and reliable screening method would be helpful in this regards especially in cost benefit selection of better plant resources of antibacterial peptides and so on (14, 15).

We used a simple agar-overlay assay which could show any antibacterial activities in the water soluble peptides and proteins of seeds. Importantly, this approach makes it possible to estimate the amount of antimicrobial activities of the peptides in a semi-quantitative manner and to detect such great antibacterial activities in *M. sativa* and *O. sativa* seeds. In screening experiments performed routinely, organic solvents are used for extraction of plant substances. The extracted materials are very complex matrices and the low concentration of effective material may be affected with unknown antagonistic or synergistic substances in the extracts. In the present study we used a buffer for extraction of antibacterial proteins and peptides. Subsequent separation of proteins and peptides on polyacryl-amide gels could help for determination of any antibacterial peptides or even proteins without antagonistic effects of other protein or peptides which could potentiate the growth of tested bacteria in routine broth or agar dilution susceptibility tests. Ammonium sulphate precipitation also confirmed the identity of antibacterial peptides in two plants.

*Medicago sativa* has been reported previously as a source of some antimicrobial substances such as saponins (16), canavanine (17) and some important antifungal defensins (15). Here we introduce *M. sativa* seeds as a source of a putative defensin (s) with great activity against 2 important human pathogens. Characterization of this peptide (s) by using 2 dimensional gel electrophoresis and LC-MS analysis is progressing in our laboratory at the moment.

The proposed screening method can be used for screening of large number of different plant seeds and even other parts of plant bodies, regarding some necessary modification in total water soluble protein extraction steps.
